# Investigating urachal carcinoma for more than 15 years

**DOI:** 10.3892/ol.2014.2502

**Published:** 2014-09-04

**Authors:** DUQUN CHEN, YIFAN LI, ZUHU YU, ZHENGMING SU, LIANGCHAO NI, YAOTING GUI, SHANGQI YANG, BENTAO SHI, YONGQING LAI

**Affiliations:** 1 Department of Urology, Anhui Medical University, Hefei, Anhui 230032, P.R. China; 2 Department of Urology, Peking University Shenzhen Hospital, Shenzhen, Guangdong 518036, P.R. China; 3 The Guangdong and Shenzhen Key Laboratory of Male Reproductive Medicine and Genetics, Institute of Urology of Shenzhen PKU-HKUST Medical Center, Peking University Shenzhen Hospital, Shenzhen, Guangdong 518036, P.R. China; 4 Department of Urology, Shantou University Medical College, Shantou, Guangdong 515041, P.R. China; 5 Department of Urology, Peking University First Hospital, Institute of Urology, Peking University, Beijing 100034, P.R. China

**Keywords:** urachal carcinoma, survival, tumor grade

## Abstract

Urachal carcinomas are rare bladder malignances, which usually present at an advanced stage with a high risk of distant metastases and a poor prognosis. To improve understanding of this uncommon carcinoma, a retrospective review was conducted for the cases observed at Peking University Shenzhen Hospital and Peking University First Hospital. The clinical outcomes were analyzed for 17 patients with a diagnosis of urachal cancer, who were admitted to Peking University Shenzhen Hospital (Shenzhen, China) and Peking University First Hospital (Beijing, China) between 1998 and 2013. The TNM staging system was used to predict outcomes. Among the 17 study patients, there were 10 males and seven females, with a median age at diagnosis of 50 years. A total of four (23%) patients presented with lymph node or distant metastasis. The median overall survival time for all stages was 57.6 months, with five patients (38.4%) alive for more than five years following treatment. The application of the TNM staging system demonstrated a median survival time of 6.2 years for stage I/II patients, compared with a median survival of 1.8 years (log-rank, P<0.001) for patients with advanced disease (stages III and IV). In addition, no significant correlation was observed between tumor size and age, and survival. In conclusion, urachal carcinomas are usually locally advanced at presentation. Surgical excision remains the predominant choice of treatment and lymph node dissection is not required unless lymph node involvement is confirmed by preoperative examination. The current results indicated that the most significant predictor of prognosis was the tumor grade.

## Introduction

During development, the fetal bladder is connected to the allantois via the three-layer canal known as the urachus. In the fifth month of development, the bladder descends causing the urachus to become stretched and a loss of the lumen (1). Subsequently, the urachus forms the median umbilical ligament. A number of abnormalities of varying significance may arise if this process is interrupted, including urachal carcinoma, which is a lethal disease that remains dormant until adulthood (2). However, urachal malignancies are rare and account for 0.5–2% of all bladder malignancies worldwide (3). Consequently, the majority of the current knowledge surrounding the disease originates from case reports and a few series performed at selected medical centers (4,5). Prognostic is often poor due to the advanced stage of the disease at diagnosis.

The current study reviewed all the cases of urachal carcinoma that were diagnosed and treated at the Peking University Shenzhen Hospital (Shenzhen, China) and Peking University First Hospital (Beijing, China) between 1998 and 2013, and describes the experience of dealing with this rare neoplasm.

## Patients and methods

Following obtaining approval from the Institutional Review Board of Peking University Shenzhen Hospital, the records of 17 adult patients with urachal carcinoma investigated at Peking University Shenzhen Hospital and Peking University First Hospital between 1998 and 2013 were retrospectively evaluated. All available clinical, laboratory, radiographic, treatment and pathological results for each patient were reviewed. The TNM (American Joint Commission on Cancer) staging system (6) was used to determine prognosis due to its consistency and universal application. Kaplan-Meier survival curves were calculated to analyze the survival data. Written informed consent was obatined from all patients.

## Results

### Patient presentation

A review of the medical records of the 17 cases of urachal cancer demonstrated that the median age of presentation was 50 years (range, 36–77 years). Males represented the majority of cases, with a male to female ratio of 1.42:1. Hematuria was the most common symptom that prompted the patient to seek medical attention, and was the predominant finding on presentation (82.3%). In two cases, the patient presented with a palpable mass in the lower abdomen, and one with dysuria (Table I).

### Imaging findings

All 17 patients underwent ultrasound (US) and the most common finding was a mass observed at the dome or frontier wall of the bladder (94.1%). In addition, almost half of the masses (47.1%) were found to be hypoechoic (Table II). Positive urine cytology results were observed for one of the five patients tested (20%). Computed tomography (CT) scans were performed in 15 patients and, among these patients, the majority of the masses (93.3%) were identified to be solid, with only one case (6.7%) presenting with a cystic mass. Calcification was observed in seven of the 15 cases (46.6%) and two of the 15 cases (13.3%) exhibited lymph node metastasis. Thickening of the bladder dome and necrosis were also observed in one of the 15 patients (6.7%). Cystoscopy was another prevalent diagnostic test; this procedure was conducted in 16/17 patients (94.1%). All mass lesions could be visualized using this technique, with the exception of one case (6.3%). Chest X-ray also revealed metastasis involving the lungs in one out of 17 patients (5.9%). The median tumor diameter of all 17 patients was 4.0 cm (range, 1.8 – 7.2 cm).

### Treatment

With the exception of one patient with confirmed metastasis who received conservation treatment, 16 patients underwent surgical excision. Extended partial cystectomy with en bloc resection of the entire urachus, including the umbilicus and the posterior rectus fascia, was the main surgical approach (75%), and pelvic lymph node dissections were performed in five of these surgeries (Table III).

### Tumor staging and pathology

Sheldon (6) and TNM (6) staging systems were used to determine tumor staging (Table IV). According to the TNM staging system, seven cases were defined as low grade and 10 as high grade. A review of the pathology reports, which were available for 16 patients, revealed that adenocarcinoma was the predominant type of tumor (87.5%), the majority of which were mucin-producing (75%) (Table V). Two patients presented with a pure transitional cell carcinoma. Tumors with mixed histology (transitional cell carcinoma adenocarcinoma) and signet ring cell adenocarcinoma were also observed. Of the five patients who underwent lymph node dissection, two patients exhibited lymph node invasion, which corresponded with the CT findings. A positive surgical margin was observed in one case.

### Survival

**Figure 1:**
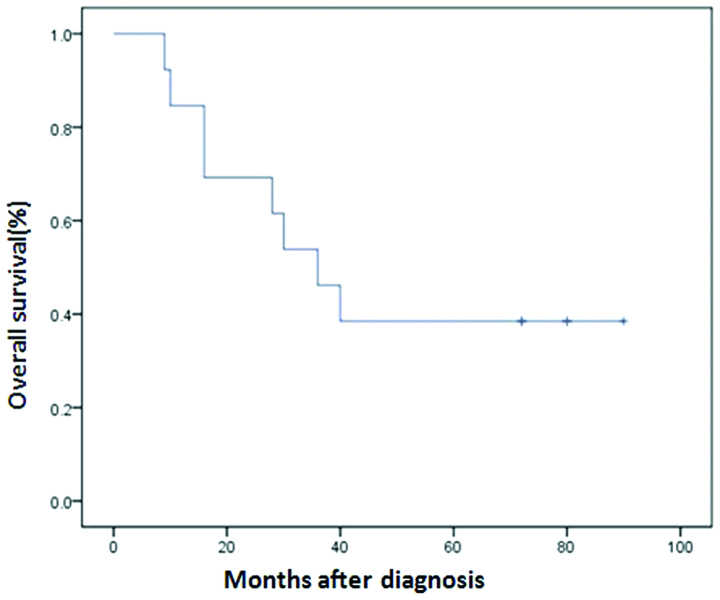
Overall survival for urachal carcinoma patients.

**Figure 2:**
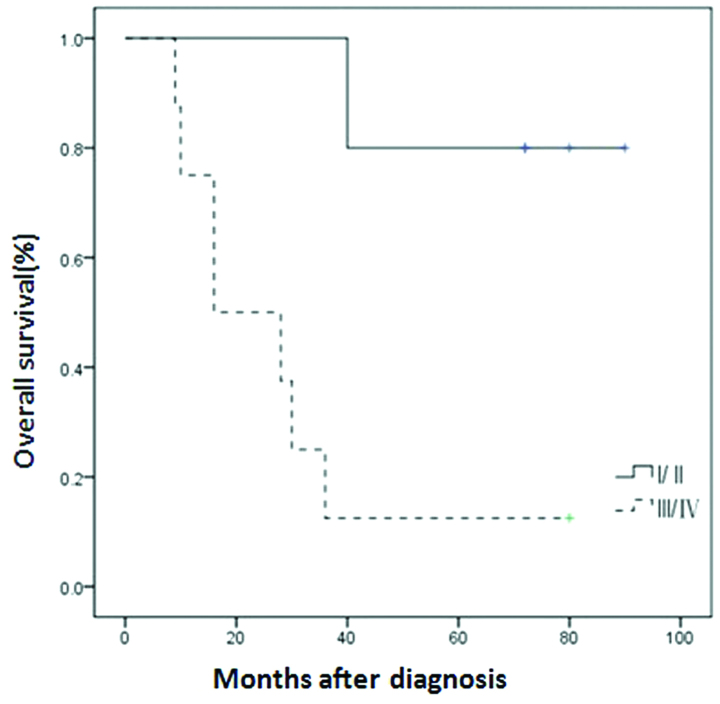
Overall survival of urachal carcinoma patients according to Sheldon tumor stage.

**Figure 3:**
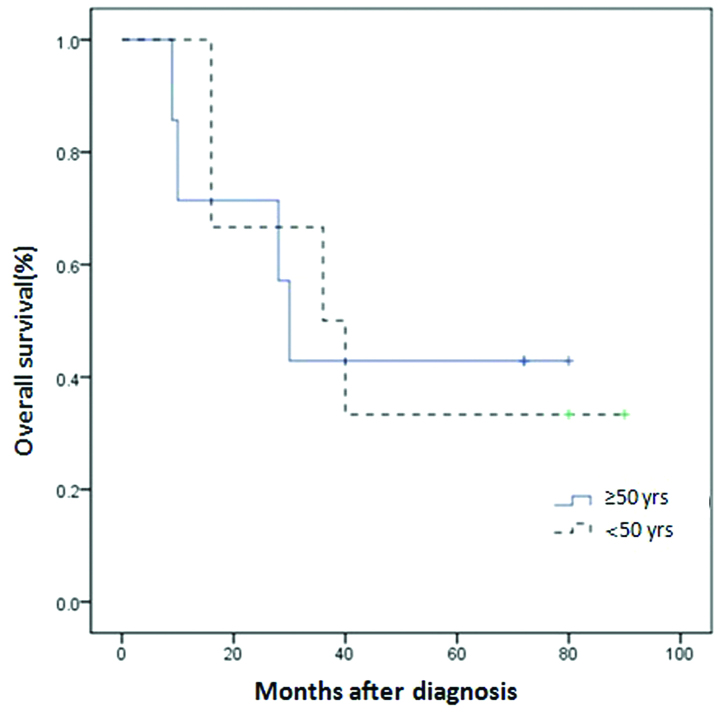
Overall survival of urachal carcinoma patients according to age.

Survival data were available for all patients. Four patients who were diagnosed later than 2008 were excluded for survival analysis. The median overall survival time for all stages was 57.6 months, with five patients (38.4%) alive for more than five years following treatment (Fig. 1). Using the TNM staging system, the differences in survival were analyzed between local disease and stage II, as well as between locally advanced and metastatic disease in stages III and IV. The results demonstrated that the median survival time for stage I/II patients was 6.2 years compared with a median survival of 1.8 years (log-rank, P<0.001) for patients with advanced disease (stage III or above) (Fig. 2 and Table VI). In addition, tumor size and age showed no significant correlation with survival (Figs. 3 and. 4).

## Discussion

The urachus usually involutes prior to birth and remains in adults as the median umbilical ligament (7). Urachal anomalies in children often present incidentally or with relatively benign symptoms (8), whereas in a considerable portion of adults, urachal anomalies present as urachal carcinomas, a rare but aggressive cancer with a poor prognosis (8).

Urachal carcinoma predominantly affects patients between 40 and 70 years old and has a male predilection. The male to female ratio in this series was 1.42:1, which is similar to that observed in other reported series (9,10). No differences have been observed in the clinical/pathological characteristics or in the prognosis of males versus females (5). Hematuria, dysuria and a palpable mass have been identified to be the most frequent symptoms (1).

US can provide a general impression of the lesion, for example the size of the mass and location, as well as calcification, as observed in the current study. CT is used to confirm the US findings and provide further information with regard to the local extent of the disease, pelvic lymph node involvement and systemic metastases. Similar to other mucinous adenocarcinomas, urachal carcinomas may also produce calcifications, which occur in 50–70% of cases and are considered almost pathognomonic for urachal adenocarcinoma (7).

With the exception of patients with metastases to distant sites, the recommended treatment is primarily surgical, with extended partial cystectomy and en bloc excision of the urachal mass, urachal tract and umbilicus. Removal of the adjacent organs is also required if they are involved with the cancer tissue (3). In the current series, two patients underwent surgery without umbilectomy, and relapsed two and six years following surgery. Ashley *et al* (11) also validated a poorer survival rate for patients who failed to undergo umbilectomy. Furthermore, the authors presented the hypothesis that negative surgical margin status is one of the most significant predictors of prognosis, which was then supported by Herr *et al* (12).

Bruins *et al* (9) demonstrated that there was no significant difference in survival between patients who underwent pelvic lymph node dissection and those who did not undergo lymphadenectomy. This was substantiated by the cases presented in the current study. With the exception of two cases with confirmed lymph node involvement by preoperative CT and pathology, the remaining three patients who underwent pelvic lymph node dissection were identified to be negative for lymph node involvement, as confirmed by pathological verification. Therefore, we recommend that lymph node dissection is not necessary unless lymph node involvement has been confirmed by preoperative examination. Patients with localized disease may respond well to surgery when umbilical resection is performed and negative surgical margins are pursued. If this is the case, partial cystectomy provides outcomes comparable to radical cystectomy (9).

In 1984, Sheldon proposed a staging system for urachal cancers (Table IV) (6). However, the Sheldon staging system does not account for the fact that urachal tumors may occur at any part of the urachus from the umbilicus to the bladder, and that these extravesical cancers may invade the bladder (6). As discussed, hematuria is the predominant symptom for patients presenting with urachal carcinoma when initially seeking medical attention, which may be a sign of bladder invasion. According to the Sheldon staging system, a high proportion of patients were classified as high grade (IIIA or above) in the this study, as only two cases were classified as low grade. The TNM staging system was implemented and confirmed that the anatomic location may be extrapolated to prognosis.

In this study, the median overall survival time for all stages was 4.8 years. The determination of prognosis based on the TNM staging system demonstrated that when the tumor is confined to the urachus itself (stage II), the median overall survival time is 6.2 years. However, a significant reduction in life expectancy was observed if the patient exhibited involvement of the regional lymph nodes or distant metastasis. The median overall survival time for patients with distant metastatic disease was almost one year. Patients with early-stage disease (stage II) exhibited a statistically significant difference in survival (P<001) compared with those with late-stage disease (stage III or IV) (Fig. 2 and Table VI), confirming the significance of the TNM system applied in urachal cancer. By contrast, tumor size and age exhibited no significant correlation with survival.

In conclusion, urachal carcinomas are usually locally advanced at presentation with a high risk of distant metastases. Surgical excision with extended partial cystectomy, en bloc excision of the urachal mass, urachal tract and umbilicus is recommended. Lymph node dissection is not necessary unless lymph node involvement has been confirmed by preoperative examinations. The current results indicate that the most significant predictor of prognosis is the tumor grade. In addition, the present study may aid in the diagnosis and management of this rare tumor.

## Figures and Tables

**Figure 4:**
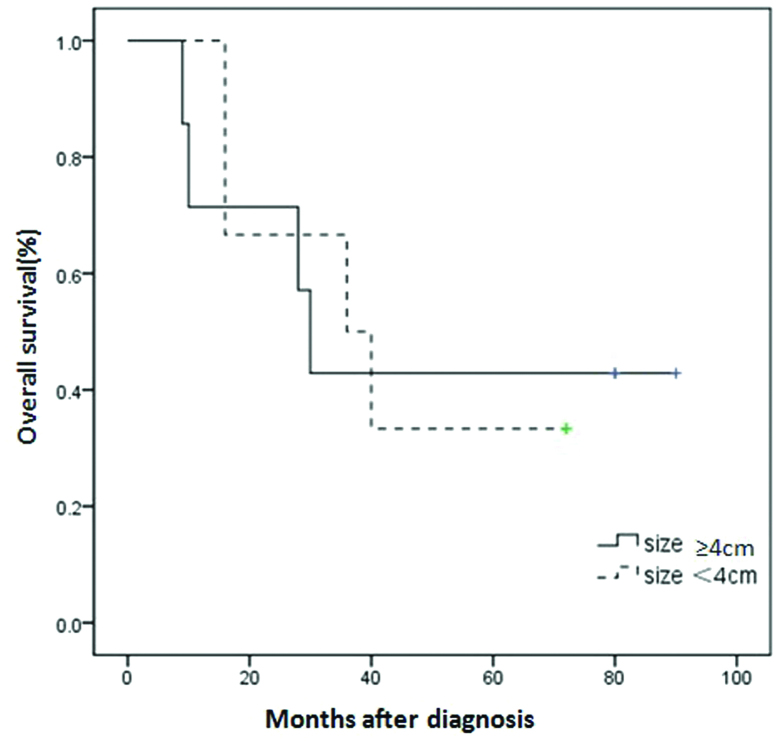
Overall survival of urachal carcinoma patients according to tumor size.

**Table I:** Clinical features of the patients at diagnosis.

Variables	n
n	17
Gender	
Male	10
Female	7
Age, years	
<50	8
≥50	9
Primary symptoms and signs	
Hematuria	14
Dysuria	1
Palpable mass	2

**Table II:** Imaging findings.

Variables	n
Ultrasound	17
Hypoechoic mass	8
Hyperechoic mass	4
Heterogeneous mass	5
Urine cytology	5
Negative	4
Positive	1
Computed tomography	15
Solid mass	14
Cystic mass	1
Thickened bladder dome	1
Necrosis	1
Calcification	7
Lymph nodes metastasis	2
Cystoscopy	16
Mass lesion visualized	15
Normal examination	1
Tumor diameter, cm	17
≥4	8
<4	9

**Table III:** Primary treatment modality.

Variables	n
n	17
Observation	1
Urachus excision	1
Transurethral bladder tumor resection	1
Radical cystectomy	2
Partial cystectomy + urachus excision	7
Partial cystectomy + urachus excision + pelvic lymph node dissection	5

**Table IV:** Urachal tumor staging and grade according to TNM staging.

Variables	n
Sheldon tumor stage	
I^a^	0
II^b^	2
III^c^	11
IV^d^	4
TNM staging system	
T1N0M0	0
T2N0M0	7
T3N0M0	6
T4N1M0	2
T4N0M1	2
Tumor grade	
Low (grades 1 or 2)	7
High (grades 3 or 4)	10

a Confined to the urachal mucosa;

b invasion confined to the urachus;

c extended to the bladder, abdominal wall, peritoneum and viscera other than bladder;

d metastatic urachal cancer to the lymph nodes of distant sites.

TNM, tumor, node, metastases.

**Table V:** Pathology of urachal carcinoma.

Variables	n
n	16
Histology	
Adenocarcinoma	
Mucin producing adenocarcinoma	9
Signet ring cell adenocarcinoma	3
TCC	2
Adenocarcinoma + TCC	2
Lymph node dissection	5
Positive	2
Negative	3
Positive surgical margin	1

TCC, transitional cell carcinoma.

**Table VI:** Prognosis according to stage of carcinoma.

Tumor stage	Median overall survival time, years
I	NA
II	6.2
III	2.4
IV	1.1

NA, not applicable.
